# Supplementary material for: Association between oral microbiome and seven types of cancers in East Asian population: a two-sample Mendelian randomization analysis
Source: Front Mol Biosci. 2023 Nov 21;10:1327893. doi: 10.3389/fmolb.2023.1327893 (PMC10702768; doi:10.3389/fmolb.2023.1327893)
Supplement: Supplementary file 1 [file DataSheet1.ZIP › Supplementary Materials/MR plots for tongue/tongue═╝/Ovarian cancer/pheno.1423_to_ovarian cancer_leave_one_out.pdf]

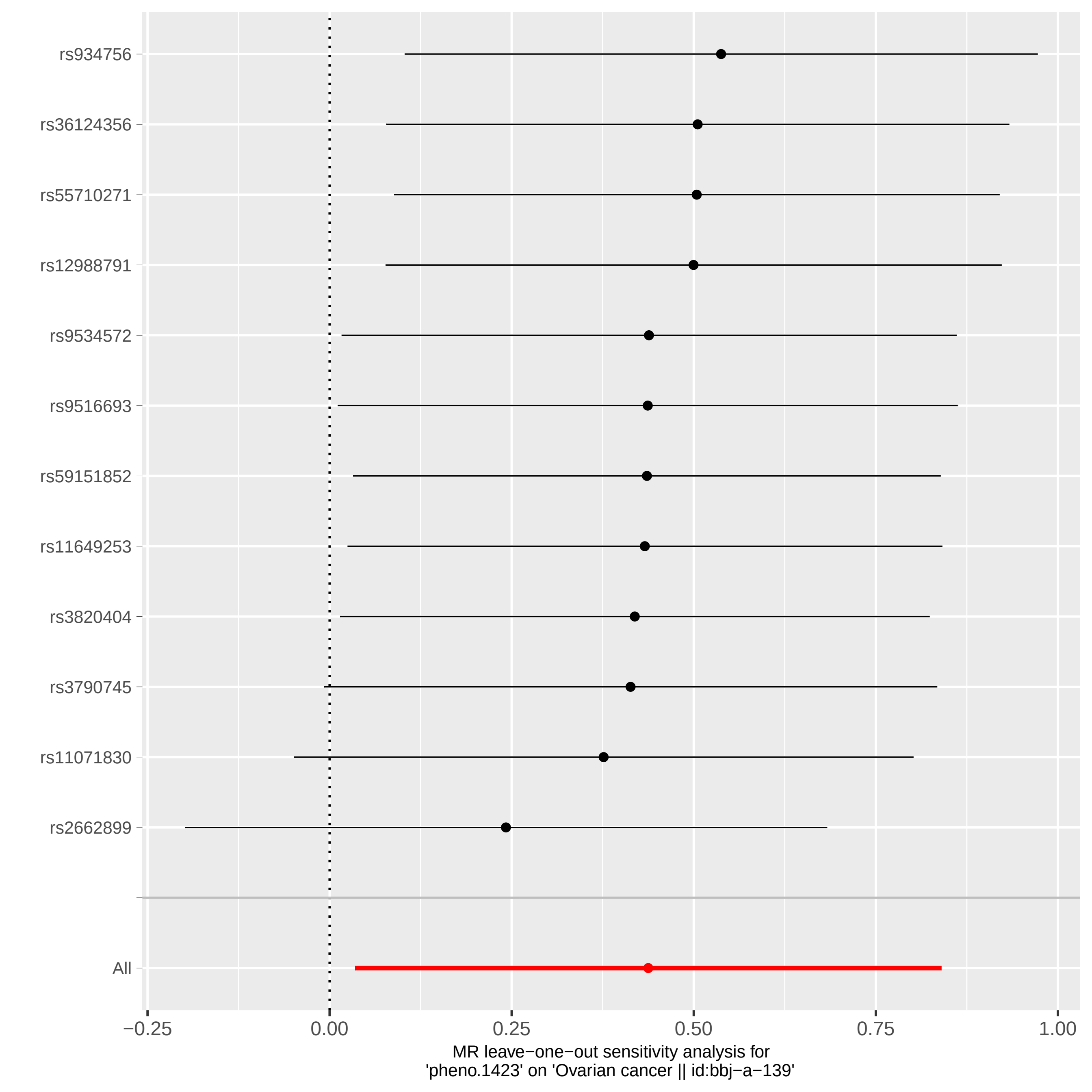

rs934756
rs36124356
rs55710271
rs12988791
rs9534572
rs9516693
rs59151852
rs11649253
rs3820404
rs3790745
rs11071830
rs2662899
All
−0.25
0.00
0.25
0.50
0.75
1.00
MR leave−one−out sensitivity analysis for
'pheno.1423' on 'Ovarian cancer || id:bbj−a−139'
